# Early Detection of Fetal Health Conditions Using Machine Learning for Classifying Imbalanced Cardiotocographic Data

**DOI:** 10.3390/diagnostics15101250

**Published:** 2025-05-15

**Authors:** Irem Nazli, Ertugrul Korbeko, Seyma Dogru, Emin Kugu, Ozgur Koray Sahingoz

**Affiliations:** 1 Biomedical Engineering Department, Biruni University, Istanbul 34015, Turkey; iremnazzlii@gmail.com (I.N.); ertugrulkorbekobme@gmail.com (E.K.); 2 Computer Engineering Department, Bursa Technical University, Bursa 16310, Turkey; seyma.dogru@btu.edu.tr; 3 Software Engineering Department, TED University, Ankara 06420, Turkey; 4 Computer Engineering Department, Biruni University, Istanbul 34015, Turkey; osahingoz@biruni.edu.tr

**Keywords:** cardiotocography, fetal health, machine learning, medical diagnostics

## Abstract

**Background:** Cardiotocography (CTG) is widely used in obstetrics to monitor fetal heart rate and uterine contractions. It helps detect early signs of fetal distress. However, manual interpretation of CTG can be time-consuming and may vary between clinicians. Recent advances in machine learning provide more efficient and consistent alternatives for analyzing CTG data. **Objectives:** This study aims to investigate the classification of fetal health using various machine learning models to facilitate early detection of fetal health conditions. **Methods:** This study utilized a tabular dataset comprising 2126 patient records and 21 features. To classify fetal health outcomes, various machine learning algorithms were employed, including CatBoost, Decision Tree, ExtraTrees, Gradient Boosting, KNN, LightGBM, Random Forest, SVM, ANN and DNN. To address class imbalance and enhance model performance, the Synthetic Minority Oversampling Technique (SMOTE) was employed. **Results:** Among the tested models, the LightGBM algorithm achieved the highest performance, boasting a classification accuracy of 90.73% and, more notably, a balanced accuracy of 91.34%. This superior balanced accuracy highlights LightGBM’s effectiveness in handling imbalanced datasets, outperforming other models in ensuring fair classification across all classes. **Conclusions:** This study highlights the potential of machine learning models as reliable tools for fetal health classification. The findings emphasize the transformative impact of such technologies on medical diagnostics. Additionally, the use of SMOTE effectively addressed dataset imbalance, further enhancing the reliability and applicability of the proposed approach.

## 1. Introduction

The widespread occurrence of health complications during pregnancy represents a significant global health concern, particularly in developing and underdeveloped countries [1,2]. These complications often result in fetal morbidity and mortality. Fetal death is clinically characterized as the loss of a fetus from human reproduction, regardless of gestational age, and not including medically induced terminations, occurring prior to the fetus being fully expelled or removed from the mother’s body. This conclusion arises from the lack of essential vital signs, such as breathing, heart function, umbilical cord pulsations, and voluntary muscle movements, as noted right after the fetus is delivered or removed. To improve maternal and fetal health outcomes, particularly in settings with limited resources where accessing quality prenatal care might be challenging, it is crucial to address the underlying causes and mitigating factors linked to fetal death [3,4,5].

**Figure 1:**
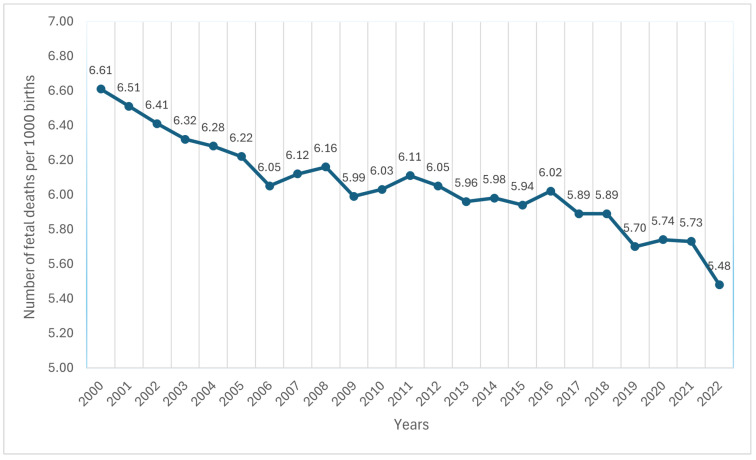
Fetal morbidity in the United States from 2000 to 2022 [7].

Fetal mortality, defined as fetal death at any stage of pregnancy, remains a significant yet often overlooked public health concern. Public attention largely focuses on infant death, which is partly due to limited awareness of fetal mortality causes and prevention methods [6]. Figure 1 presents fetal fatality statistics in the United States from 2000 to 2022 [7]. Monitoring fetal health is crucial for maternal and neonatal well-being [8]. Cardiotocography (CTG), a widely used clinical tool for nearly 60 years, enables the early detection of fetal impairment by recording fetal heart rate (FHR) and uterine contractions (UCs). The evaluation of FHR and UCs on CTG includes a total of 21 attributes. These attributes help obstetricians determine the fetus’ health. Traditionally, fetal health assessment relies on manual clinical evaluations, which are prone to human error [9]. To enhance accuracy, this paper utilizes CTG data and machine learning (ML) algorithms [10], offering healthcare professionals a more reliable diagnostic tool. Successful implementation would advance fetal monitoring technology and improve fetal health assessment.

Ensuring accurate fetal health assessment through CTG and ML relies on high-quality data, specifically with balanced datasets. However, real-world datasets are often inherently imbalanced because of the natural distribution of events or phenomena being studied. For example, in medical diagnostics, diseases such as cancer or rare genetic disorders occur much less frequently in the population than healthy cases, resulting in the representation of the minority class. This imbalance arises because certain events are naturally infrequent or harder to capture. Similarly, the dataset on fetal health is also imbalanced, and most studies have not adequately addressed this imbalance as a primary concern.

The balanced distribution of a dataset is critical in ML-based classification, as it directly affects the model’s fairness, performance, and interpretability [11]. When a dataset is imbalanced, with one class significantly outweighing others, the model tends to become biased toward the majority class. This often leads to misleadingly high accuracy, as the model predominantly predicts the majority class while neglecting minority classes. Such biases can severely compromise the generalization of the model, particularly for applications where the accurate detection of minority instances is crucial, such as healthcare diagnostics or fraud detection. Additionally, metrics like precision, recall, and F1-score become more reliable and meaningful when classes are well represented, as balanced datasets prevent skewed assessments of the model’s performance.

Balanced datasets also promote better optimization and interpretability. Models trained on evenly distributed data tend to converge more effectively, as training processes are not overly influenced by the majority class. This ensures that the model learns to distinguish features across all classes equitably, resulting in a more robust and stable classifier. Furthermore, balanced data improves interpretability by allowing all classes to contribute proportionally to the decision-making process, enabling clearer insights into the strengths and weaknesses of the model. When natural balance is unattainable, techniques such as resampling, class weighting, or data augmentation can be employed to mitigate the imbalance, ensuring the model’s reliability and fairness across diverse scenarios.

The remainder of this paper is organized as follows: Section 2 provides a comprehensive review of the existing literature on fetal health classification systems and highlights the advancements in ML applications in this domain. Section 3 presents the proposed approach by outlining the workflow of the models, beginning with an overview of the dataset. It then details the data processing techniques, the algorithms utilized, and the application of the Synthetic Minority Oversampling Technique (SMOTE) to mitigate challenges associated with imbalanced datasets. Section 4 presents the experimental results, including a thorough evaluation of performance metrics to assess the effectiveness of the proposed system and discuss the results. Finally, Section 5 concludes this paper by summarizing key contributions, discussing limitations, and suggesting potential directions for future research.

The main research objectives of this article are presented to highlight the key challenges addressed and the innovative approaches proposed, including the following:

- Train eight different ML models and a deep learning model capable of classifying the fetal health status using CTG data;
- Evaluate the model’s performance using various metrics, including balanced accuracy and the balanced error rate;
- Validate the models by using a clinical dataset under real-world conditions;
- Measure performance of proposed models while minimizing the impact of an unbalanced dataset;
- Benchmark performance against existing fetal health monitoring models;
- To ensure comprehensive documentation of the proposal’s methodology and results through detailed reports and presentations.

In many studies within the literature, the classification accuracy is often used as the main performance metric. However, this approach is not suitable for imbalanced datasets, which are commonly encountered in health-related applications. Therefore, the success of this proposal will be evaluated based on achieving improved performance metrics, such as high balanced accuracy [12], while also demonstrating clinical usability and providing clear, shareable documentation of the findings.

## 2. Literature Review

Despite advancements in prenatal care, fetal death remains a significant global challenge underscoring an urgent need for improved prevention strategies and early detection mechanisms supported by cutting-edge technologies. In recent years, efforts have increasingly focused on the early detection of fetal abnormalities through the use of decision support systems, including machine learning techniques. Creating a prenatal health classification model is essential, as it can improve efficiency and save medical resources throughout the diagnostic procedure. The most common method of detecting perinatal death and implementing early preventive actions for the health of both the mother and the fetus is a test that minimizes stress. Experts assess fetal health by examining the data generated by the non-stress test (NST), equipped with a built-in printer, in a hospital environment [13]. The NST gadget’s internal memory contains numerical data that can be labeled by ML to diagnose it. Given this information, supervised learning techniques can be employed to classify FHR patterns.

Studies on fetal health classification focus on the analysis of cardiotocographic data used in the evaluation of fetal health. Ref. [14] investigated the prediction of fetal health status from CTG data using various ML algorithms. The performance of algorithms such as SVM, RF, MLP, and KNN was evaluated, and how these algorithms could be utilized to classify fetal health status was explored. In this study, the authors tried to classify the fetal health status as either healthy, in need of further investigation, or abnormal. They also used a dataset including 1831 records collected by personnel from the University of Porto. The dataset is stored in the UCI-ML Repository. The RF algorithm and XGboost exhibited the best performance compared to others, demonstrating higher accuracy, sensitivity, recall, F1-score, and support values. These findings indicate that better results can be achieved with appropriate feature engineering on CTG data.

The results of this study showed that ML algorithms can be used effectively to predict fetal health status. In particular, the RF algorithm outperformed other algorithms. This study provides significant information on the potential use of ML algorithms in the early diagnosis of fetal health conditions.

Rahmayanti et al. examined the classification of fetal health status using ML methods based on CTG data [15]. The names of the classes were normal, suspect, or pathological. The dataset they used consists of 2126 records stored in the University of California Irvine Machine Learning Repository. According to experimental results, the LightGBM and RF algorithms produced the highest AUC and F1-score values, demonstrating the most effective classification performance (0.99 and 0.98, respectively). Although the accuracy rate of the LightGBM model was determined to be 0.99, the accuracy rate of the RF model was 0.98. These results indicate that ML algorithms can be effectively used on CTG data. In particular, LightGBM stands out with its high performance obtained through data preprocessing techniques and the optimization of algorithm parameters. The authors explained that twelve ML models were initially experimented with the CTG dataset in another study. Their experiments revealed that the Blender model performed with an accuracy rate of 0.959 and a recall rate of 0.916 compared to traditional ML models [16]. The aim of a study conducted by Miao et al. in the USA was to obtain and compare the deep neural network classification and prediction models of ML in all clinical instances, including fetal assessments in the CTG data, and to predict the multiclass morphological patterns compared to the 10 target class outcomes [17]. The dataset used by the authors includes 2126 recordings available in the UCI-ML Repository.

Kuzu and Santur introduced an ensemble learning-based approach with the CTG dataset to diagnose fetal health status [18]. The accuracy achieved by the predictor on the test dataset was above 0.995. Using the experimental results, a fetal health diagnosis can be made during NST with the help of ML. It has a higher accuracy than other approaches presented in some papers. Furthermore, the proposed approach has less computational complexity.

Various ML algorithms for classification in the field of fetal health were investigated by Regmi et al. [19]. The authors tried to classify the dataset including 2126 records as normal, suspect, or pathological. They indicated the classification performance of various ML algorithms, including SVM, RF, and attentive interpretable tabular learning, on fetal health before implementing dimensionality reduction methods such as principal component analysis and linear discriminant methods. A TabNet model on a fetal health dataset achieves a classification accuracy of 94.36%. From the results obtained from this study, the combined LDA classifier had a better classification outcome compared to the RF classifier combined with PCA, which gives the highest accuracy. Their optimum classification result was an accuracy of 91.13% for prenatal health abnormality.

Ramdas and Sumalatha carried out a study using seven ML models to diagnose fetal illness and the ensemble techniques voting and stacking classifier. The dataset they used consists of 2126 records collected by personnel from the University of Porto. Feature selection and feature extraction techniques enabled the selection of the most vital and important attributes from the dataset. To balance the dataset, the authors used ANOVA, Chi-square, PCA, ICA, and SMOTE. The SC with a meta-classifier as the RF model and Chi-square with PCA scored higher than all previously used methods, as the proposed reactor system achieved scores of 98.74%, 98.82%, 96.22%, 96.32%, and 98.64%, respectively [20].

A new ML technique for classifying fetal health using a LightGBM classifier trained on a comprehensive dataset is presented in [21], achieving an accuracy of 98.31% on the test set. This model demonstrates great potential for the early detection and treatment of fetal health problems due to its high accuracy, generalization ability, and sensitivity to abnormalities. In [22], Brzoza compares three AI systems—K-nearest neighbor, naive Bayes, and soft sets—to identify the most effective approach. However, the naive Bayes classifier yielded unsatisfactory results compared to K-nearest neighbor, with an average precision of 0.82 over 10 iterations.

Signorini et al. evaluated 15 ML approaches to distinguish between healthy fetuses and those with intrauterine growth restriction (IUGR) using a dataset of 12 physiological rhythm variables from the antepartum CTG records [23]. Testing on 60 healthy and 60 IUGR fetuses, the Random Forest method achieved the highest performance with a classification precision of 0.911, while classification trees, logistic regression, and SVM yielded comparable results. Further research emphasized generic ML methods, including artificial neural networks, SVM, extreme learning machines, and RF, applied to CTG data with two labels instead of three [24]. When five ML techniques were compared, their findings highlighted that although most methods showed satisfactory performance, artificial neural networks achieved an outstanding accuracy of 99.73% and a precision of 97.94%.

In another study [25], the researchers evaluated the efficiency for three ML models: RF Baseline, LightGBM, and CVE LightGBM. Their findings revealed that the most effective model consisted of a cross-validation ensemble of LightGBM models, achieving an impressive 95.82% AUPRC. The utilization of LightGBM with Bayesian optimization significantly enhanced the baseline performance, while the incorporation of a cross-validation ensemble led to a slight yet favorable improvement in performance.

In paper [26], the authors applied five classifiers (RF, AdaBoost, CatBoost, XGBoost, and LGBM) to find the correct class without over-sampling for CTG records. Then, they employ an ensemble of these classifiers with over-sampling way. CGT readings include 3602 records for train purposes and 1201 records for the evaluation purporses. The results of this work show that LGBM, XGBoost, and CatBoost provide 99% accuracy. The authors determine that utilizing over-sampling to balance the CTG data delivered the essential precision to the dataset, facilitating training and classification. Their ensemble models are very accurate in classifying the dataset.

Rayhan et al. constructed and compared models to assess their effectiveness in detecting fetal health. A total of five models were constructed, with each model utilizing one of the five ML techniques, incorporating all the features in the dataset used for describing variables. The other five models, however, employed the attributes chosen by the MRMR method along with the identical ML techniques. Through comparative analysis, it was found that the XGBoost algorithm-based model outperformed others in automatically detecting fetal health. Their model achieved an impressive accuracy of 96.7% and an F1-score of 96.3% in the pathologic class [27].

The researchers have designed a sophisticated ensemble model known as ETSE, which fuses the capabilities of Tuned SVM with ExtraTrees in [28]. Prior to testing seven different classifiers, a series of data preprocessing steps like purging outliers, filling in missing data, standardizing datasets, and implementing data sampling were conducted. The combination of ExtraTrees and SVM stood out, achieving an unparalleled 99.66% accuracy and registering perfect precision, recall, and F1-scores. The success of the ETSE model is heavily contingent on employing ensemble learning methods and meticulous hyperparameter optimization. Conclusively, the ETSE model is recognized as an impactful analytical tool for prenatal fetal health screening, playing a crucial role in promoting healthier infant outcomes.

Das et al. [29] introduced a machine learning model for analyzing cardiotocography data to distinguish labor stages, evaluating its performance with metrics including ROC-AUC scores using classifiers like SVM, RF, MLP, and bagging. While all classifiers performed well, SVM and RF achieved notable results, with SVM reaching 0.974 accuracy in equivocal cases and RF achieving 0.98, along with sensitivities of approximately 0.964 and specificities near 0.98, respectively. In the second labor stage, SVM and RF reported accuracies of 0.906 and 0.893. In related work, the authors of [30] evaluated ML models for predicting fetal well-being via CTG data, employing Logistic Regression, KNN, RF, and Gradient Boosting Machine (GBM), assessed by accuracy, precision, recall, and F1-score. RF achieved the highest accuracy at 0.99, further demonstrating its effectiveness with an R Square value of 0.999 and an accuracy score of 0.93, proving its higher efficiency over Logistic Regression, KNN, and GBM.

In [31], researchers have engaged in the classification of fetal states as normal, suspect, and pathological by employing an array of seven ML algorithms, namely AdaBoost, RF, K-nearest neighbors, SVM, Gradient Boosting Classifier, DT Classifier, and Logistic Regression. To ensure a fair examination of the dataset, a standardization technique known as Standard Scaler was applied. Of all the models, the gradient boost classifier demonstrated exceptional performance, attaining an accuracy of 0.95, with precision rates of 0.96 for normal, 0.85 for suspect, and 0.97 for pathological states. The recall scores associated with the states normal, suspect, and pathological were 0.98, 0.78, and 0.94 respectively, while F1-scores stood at 0.97 for normal, 0.81 for suspect, and 0.96 for pathological states. Nevertheless, it is worth noting that the RF, SVM, and KNN models obtained a perfect precision of 1.00 specifically in identifying the pathological state.

In a recent study conducted in 2023 [32], authors similarly trained ML models to identify fetal health status analyzing cardiotocogram data and focused on the explainability of these models. Using XGBoost, LightGBM, and SVM algorithms, accuracy rates of 98.79%, 99.19%, and 99.59% were achieved, indicating high classification success of these models. Additionally, using SHAP, LIME, and FAB methods, decision-making processes of the models were made explainable. With these methods, it was extensively examined on which features the models based their predictions, contributing to the understanding of the models’ decision-making mechanisms.

Dwivedi et al. [33] aimed to classify fetal health status using cardiotocography data and explain the main contributing factors. In the study conducted with machine learning (ML) and Explainable Artificial Intelligence (XAI) methods, automatic model selection and hyperparameter optimization were performed using PyCaret. The study used the SMOTE technique to balance the imbalanced datasets and provided multiple metrics to evaluate the performance of the model. In addition, the decision-making processes of the model were explained with XAI methods, and important features were determined. In this context, abnormality in short-term variations stood out as the most critical factor in fetal health assessment. The best-performing model was LGBM. Accuracy was 95.61%, Precision was 95.52%, Recall was 90.56%, F1-score was 95.5% and AUC was 98.64%.

As observed in Table 1, most of the reviewed studies prefer accuracy as the primary performance metric. However, we believe that balanced accuracy and the balanced error rate serve as more suitable comparative metrics for our analysis. Additionally, the table highlights the most commonly used machine learning algorithms. Accordingly, we have implemented these algorithms to achieve more reliable results.

Although these solutions demonstrate acceptable performance, their effectiveness is often constrained by the imbalanced nature of the datasets, which mirrors real-world distributions. To address this problem, synthetic data were generated for the minority classes to reduce the imbalance rate. Furthermore, balanced accuracy, a more suitable metric for evaluating such datasets, was prioritized in the analysis. In addition, a deep learning approach leveraging Deep Neural Network architecture was adopted to assess the impact of current technologies.

## 3. Proposed Approach

The objective of this paper is to train and compare eight ML models and a deep learning model to classify fetal health using CTG data with a high balanced accuracy rate by removing the effect of an imbalanced dataset. CTG provides valuable information on FHR and UC, which are crucial to assessing the well-being of the fetus. Traditionally, obstetricians manually analyzed CTG data and this process is a time-consuming task and prone to errors. Therefore, training an automated model as a decision support system for the classification of fetal health is essential to accelerate early diagnosis and treatment while conserving valuable medical resources. In current practice, the CTG results are printed on paper and manually interpreted by the doctor to assess fetal health. This process relies heavily on human expertise and is subject to delays and potential inaccuracies. One of the main issues with existing systems is the time-consuming process of printing and interpreting the results. Given the importance of rapid assessment and intervention, this delay can affect patient diagnosis and treatment decisions on time. As part of this effort, CTG data will be processed using advanced software tools, which will allow the extraction of relevant features and facilitate the application of ML techniques.

**Figure 2:**
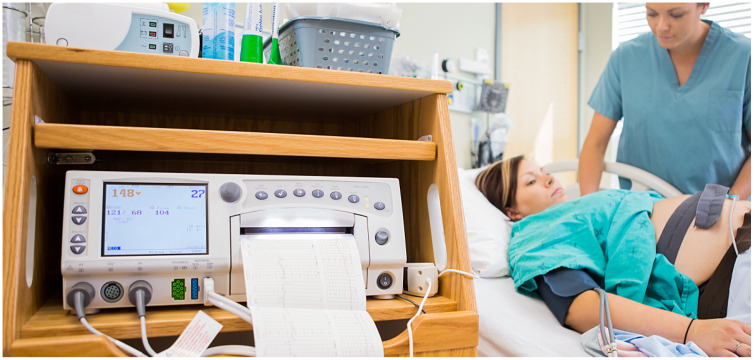
CTG Device and result of a Patient [35].

The graphical representation of CTG data, which includes FHR and UC, as shown in Figure 2, serves as a critical input for this analysis. The selection of the optimal ML algorithm will be guided by a rigorous performance assessment, ensuring that the system is effective and generalizable to diverse clinical scenarios. FHR is a crucial measure of the condition of the fetus. The fetal neurologic system regulates the fetal heart using both input and output networks. The fetal neural system directly communicates with the health of the baby by controlling the FHR. The CTG signal is used to detect the health condition of the fetus by meticulously analyzing the specific characteristics of the signal [34]. The normal heartbeat for a normal fetus ranges from 110 to 160 pulses per minute. Nevertheless, in case the baseline heart rate decreases below 110 beats per minute for a continuous period of 10 min, it is going to be categorized as Bradycardia. Conversely, if the heart rate exceeds 160 rhythms per minute for a duration of ten minutes or more, it is going to be classified as Tachycardia. The typical range of heart rate variability from the baseline is 6–25 beats per minute. Rapid increase in speed is crucial for an ideal pregnancy, while a decrease in speed is worrisome. On the other hand, the second critical CTG Data, UC, is vital for assessing fetal health, particularly during labor, as they influence oxygen supply to the fetus and help evaluate fetal stress. Monitoring UC alongside FHR via CTG aids in detecting fetal distress, understanding labor progress, and diagnosing complications like hypoxia, umbilical cord compression, or placental insufficiency. Abnormal UC patterns, such as excessive or insufficient contractions, guide interventions like oxytocin administration or cesarean delivery to mitigate risks and ensure maternal-fetal well-being.

The evaluation of CTG results definitely requires expert knowledge. However, sometimes small but important changes in CTG may not be detected by the human eye. In addition, the psychology and fatigue of the expert who will make the evaluation on that day may prevent reaching the correct result. At this stage, a mechanism is needed that will reduce human error to zero. ML emerges as a method that can offer the most appropriate solution in this regard.The detailed analysis of FHR and UC metrics, as discussed above, forms the foundation for the dataset used in this study. The dataset encompasses comprehensive CTG recordings that capture these critical parameters, enabling the application of machine learning algorithms to detect and classify fetal health conditions with precision.

### 3.1. Dataset/Model Overview

In this section, we introduce the UCI CTG dataset used in this research, which focuses on pregnancy complications. The dataset was obtained from the CTG databases available on Kaggle [36] and was generated using SisPorto 2.0 [37], a program designed for the automatic analysis of CTG results. SisPorto 2.0 provides an automated analysis of several key groups of fetal monitoring indicators, including FHR parameters, uterine contractions, fetal movements, variability measures, and histogram-based features. These categories correspond to standard clinical observations routinely used in the assessment of fetal well-being and are represented in the dataset utilized for model development. This clarification has been incorporated into the Dataset Description section of the revised manuscript.

Despite its moderate size, the UCI CTG dataset remains a valuable benchmark in fetal health classification research due to its well-structured features, reliable annotations by expert obstetricians, and clear classification labels. It consists of 2126 participants from pregnant women in their third trimester, each entry containing 21 attributes that evaluate fetal health conditions. The CTGs were classified by three specialist obstetricians and each was given a consensus classification label. The classification considered both a morphological pattern and a fetal condition (Normal, Suspect, Pathological). The attributes used to measure fetal heart rate and uterine contractions are listed in Table 2.

**Figure 3:**
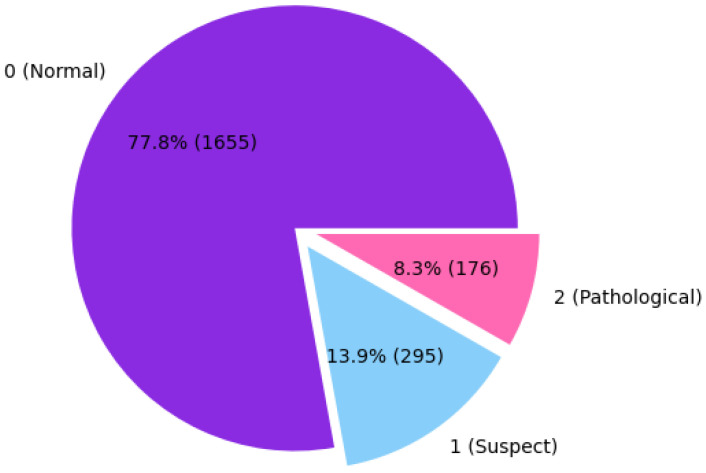
The Percentage Distributions of the Classes.

Within the dataset, the three classes, Normal, Suspect, and Pathological, are represented by the numbers 0, 1, and 2, respectively. The distribution of the dataset for each class is shown in Figure 3. To establish the accuracy of the CTG results, three professionals in the domain of gynecology classified the CTG records, and their interpretations were considered as the gold standard for classification.

### 3.2. Data Processing

This section describes in detail the method of the study and the steps to be taken. For the algorithms to perform well, it is important that the dataset undergoes some pre-processing. All data contained in the dataset must be checked. It should be detailed how you prepare the data, and it should be put in a position where every algorithm can work as much as possible (cleaning, transforming, re-sampling, etc.). The correlation between properties and, if any, the selection of properties for higher properties should be considered. After model selection is completed, the model is trained and evaluated. Parameters should be evaluated to improve the performance of the model. A user interface is developed with the highest-performance algorithm.

Firstly, the dataset was thoroughly analyzed to ensure its readiness for further processing. During this analysis, the presence of missing values was checked, and it was confirmed that the dataset contained no missing values. This finding ensured that imputation or data cleaning techniques related to missing entries were unnecessary. Additionally, the feature with the longest name, “percent time with abnormal long term var”, was identified. For the sake of clarity and to maintain concise representation, this feature name was shortened to improve readability and usability in subsequent analyses. The overall distribution of the dataset was examined, as depicted in Figure 3. This visualization provided an overview of the data structure and helped identify any potential anomalies or patterns. Furthermore, the distribution of the key features was analyzed in greater detail using histogram charts, which offer insights into the variability and tendencies of the data. These histograms are presented in Table 2, highlighting the characteristics of the most significant attributes in the dataset.

**Figure 4:**
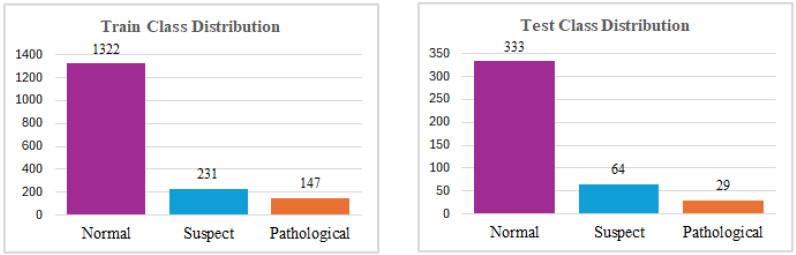
The Distribution of Training and Testing Dataset for ANN.

After carefully analyzing the graphical representations of the data, it was observed that the number of instances in each class was significantly imbalanced. Its limited size and imbalanced distribution pose significant analytical challenges. To address this issue, it is essential to ensure that the class distributions are balanced, as this is a crucial prerequisite for training a reliable and predictive model. To minimize redundancy and potential multicollinearity issues, the ‘histogram mean’ feature was excluded from further analysis. Following feature selection, the dataset was partitioned into training and testing subsets using the standard split method. In this process, 80% of the data was allocated for training the model, while the remaining 20% was reserved for evaluating its performance on unseen data. This split ensures that the model is trained on a substantial portion of the data while retaining enough samples for a robust evaluation. The distribution of data across the training and testing sets after the split process is depicted in Figure 4. This visualization provides insights into how the data is represented in each subset, ensuring that no significant biases or anomalies were introduced during the splitting procedure.

**Figure 5:**
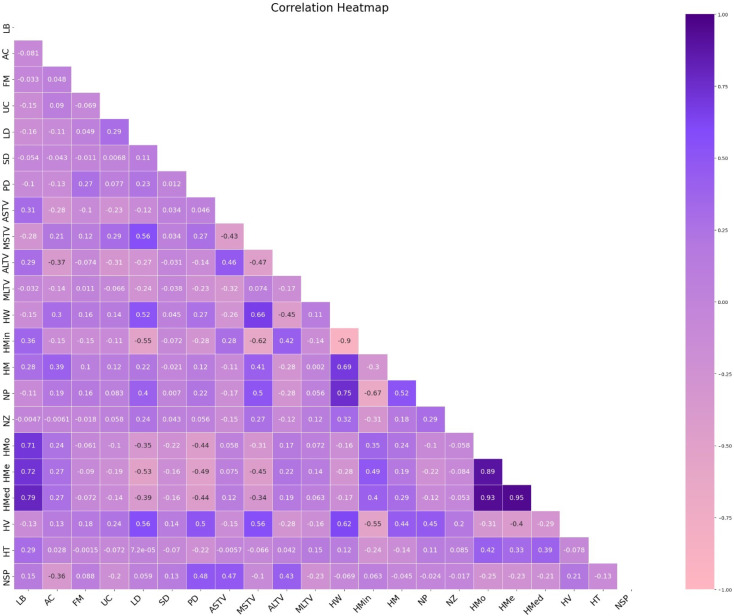
The Correlation Matrix of the Features.

As illustrated in Figure 5, a correlation matrix of the features was constructed to identify the relationships between them. Based on this analysis, it was observed that the ‘histogram mean’ feature exhibited a high degree of correlation with other features.

### 3.3. Importance of the Features

Understanding the order of feature importance in a dataset is fundamental for training robust, efficient, and interpretable machine learning models. By analyzing feature importance, data scientists, system developers, and researchers can determine the variables that contribute the most to the predictions of a model, enabling more informed decision-making capability in feature selection and the design of complex models. This process not only enhances the performance of the models, but also reduces computational costs of the system by eliminating redundant or irrelevant features, leading to faster training times and improved generalization to unseen data. In this paper, our aim is not to decrease the number of features; instead, understanding the most important features in the context of explainable AI is our focus. Furthermore, identifying the most influential features helps mitigate the risk of overfitting because models trained on excessive, noisy, or less informative variables tend to capture spurious patterns rather than meaningful relationships.

**Figure 6:**
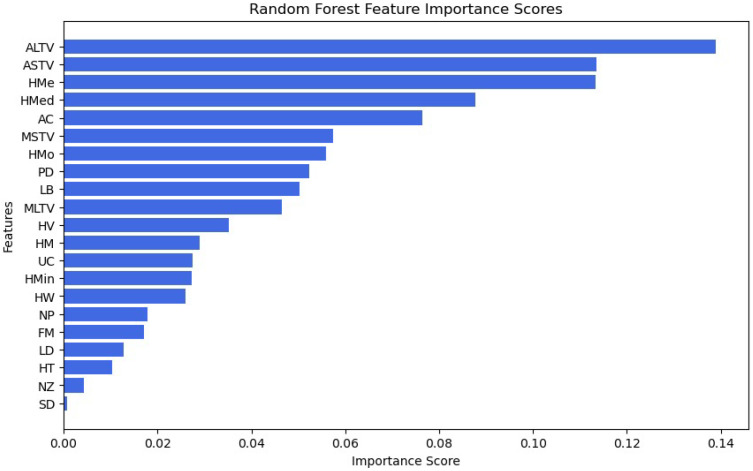
Feature Importance Scores.

In the literature, there are several techniques that assess the importance of the features, each offering different perspectives on how the features impact the predictions of the model. Methods such as the importance of permutation evaluate how random shuffling of a specific feature affects model performance, providing a measure of its contribution. Tree-based approaches, including feature importance scores from algorithms like Random Forest, ExtraTrees, and Gradient Boosting, inherently rank features based on how frequently they are used in decision splits. In our work, we used the Random Forest algorithm to understand the order of importance of features as in Figure 6.

Feature importance analysis is particularly valuable in fields where interpretability and trust in model decisions are critical. In healthcare, for instance, understanding which features influence fetal health predictions can improve diagnostic accuracy and guide medical decision-making. As seen from the figure, the most important features are Percentage of Time with Abnormal Long-Term Variability, Percentage of Time with abnormal short-term variability, and Histogram Mean. Ultimately, understanding the importance order of features allows machine learning practitioners to build models that are not only more accurate and efficient, but also more transparent and interpretable, fostering trust and usability in real-world applications.

### 3.4. Workflow of the Proposed Model

**Figure 7:**
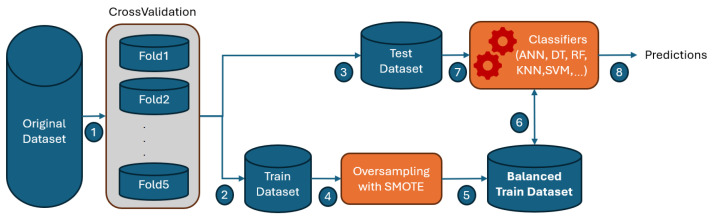
Workflow of the Proposed Model.

The proposed model for early detection of fetal health conditions leverages a systematic machine learning pipeline designed to address the challenges of classifying imbalanced cardiotocographic data. The workflow of the proposed model incorporates cross-validation to ensure robust model evaluation and employs the synthetic minority sample technique (SMOTE) to mitigate the effects of class imbalance in the dataset as depicted in Figure 7. By combining this pre-processing strategy with a diverse set of machine learning classifiers, the model aims to improve the prediction accuracy and reliability of fetal health classification. The following steps outline the detailed workflow, from data preparation and oversampling to classifier training and prediction generation, emphasizing its adaptability to efficiently analyze CTG data.

Step 1: The process begins with the original dataset, which serves as an input for training and testing.

Step 2: The dataset is divided into multiple subsets or folds (e.g., Fold1, Fold2, …, Fold5) for cross-validation. Each fold is used once as the test set, while the remaining folds are used as the training set.

Step 3: A specific fold is designated as the test dataset during each cross-validation iteration.

Step 4: The remaining folds are combined to form the train dataset.

Step 5: The training dataset undergoes oversampling using SMOTE to address class imbalance by generating synthetic samples for the minority class.

Step 6: The output of SMOTE is a balanced training dataset with equal representation of classes.

Step 7: Machine learning classifiers (e.g., ANN, Decision Trees, Random Forests, KNN, SVM, and others) are trained on the balanced train dataset.

Step 8: The trained classifiers are evaluated on the test dataset to generate predictions, which can be used to assess model performance.

This workflow ensures robust model evaluation by leveraging cross-validation while addressing class imbalance through SMOTE.

### 3.5. Algorithms and Techniques

In this study, a comprehensive analysis of ML algorithms was performed to create an effective system for fetal health classification. Eleven widely used algorithms, spanning different ML paradigms, were selected to evaluate their performance in the CTG dataset. Selection covers a diverse range of techniques, including DT, ensemble methods, probabilistic models, and instance-based learning, ensuring a robust comparison of their classification capabilities. Each algorithm was implemented and fine-tuned to optimize its performance, and the results were analyzed to identify the most suitable approach for this critical healthcare application. The following parts detail the techniques and characteristics of these algorithms and how they were applied in the context of this study.

**Decision Tree (DT)** and their ensembles, known as forests, are extensively researched and highly used tools in the domains of ML and data science. These concepts are included in almost all introductory courses on these topics, and there are numerous surveys that cover the substantial literature on them [38]. DT is a form of supervised learning approach that is used for problems such as regression and classification. A DT is a graphical representation of choices and their potential outcomes, which considers random events, costs of resources, and overall value.

Regarded as one of the top-performing models in machine learning, **Random Forest (RF)** represents a modification of bootstrap aggregating, building a large ensemble of trees and then averaging their results.As is customary when dealing with continuous response variables, the criterion used for least-square splitting is employed in the construction of each tree [39] Combining tree predictors in a way that each tree relies on a randomly distributed vector with the similar distribution over all trees in the forest is called a RF. As the number of trees in a forest grows larger, the generalization error converges to a limit. In a forest of tree classifiers, the overall generalization error is proportional to the strength and correlation of each individual tree [40].

**K-Nearest Neighbor (KNN)**, is a simple, non-parametric ML algorithm used for classification and regression tasks, and it has an easy-to-implement framework and produces good classification results. Considering that it does away with the need for neural networks to undergo training [41]. It is part of supervised learning and is widely used in pattern recognition, data mining, and unauthorized entry detection. Due to its non-parametric nature, this method is commonly used only once in real-life situations, without making any basic assumptions about the distribution. Users have been provided with initial data that categorizes coordinates into groups based on a specific characteristic. This approach involves assigning data to a certain group based on the observation of which group its closest neighbors belong to.

**Support Vector Machines (SVMs)** are a type of machine learning algorithm designed to maximize the margin of separation between classes by identifying a hyperplane in a multidimensional feature space. A key advantage of SVMs is their ability to handle nonlinear and non-separable data by transforming it into a higher-dimensional space using the kernel method, making them more robust and flexible compared to logit or probit link functions [42]. **CatBoost**, short for “Categorical Boosting”, is an open-source machine learning technique developed by Yandex, widely used in R (version 4.0 and later) and Python (version 3.5 and later) [43]. It employs a Gradient-Boosting Decision Tree (GBDT) framework with symmetric decision trees as base learners to efficiently process categorical features while minimizing gradient bias and prediction shift, thus enhancing precision and reducing overfitting [44].

A popular tree-based model is **Light Gradient Boost Trees (LightGBM)** which is a technique for ML that is designed for use with large amounts of data. To minimize loss, it employs gradient descent optimization and iterative weight changes. In addition to its extraordinary speed and accuracy, it also possesses several distinctive characteristics, such as a histogram-based approach for best-split spots, a leaf-wise branching strategy, and a bespoke data storage arrangement [45].

**Gradient Boost Trees** is a popular approach in ML, which stands for Gradient Boosted Trees and is known for its strength and flexibility. Additionally, it can be utilized for difficulties involving categorization as well as regression. The GBT is an ensemble of DTs that tries to develop a model step-by-step in order to enhance prediction performance. One of the most widely used statistical learning strategies, gradient boosted trees produced 17 winning entries out of 29 in 2015’s Kaggle competitions [46]. The fundamental concept of gradient boosting is based on the idea of fitting a series of connected fewer effective learners. Only a small amount of variation that was not caught by the trees that preceded it is explained by each tree [47].

Finally, as one of the most popular ML models, **Artificial Neural Networks (ANNs)** are computational models inspired by the structure and functioning of the human brain. They consist of interconnected layers of nodes (neurons) that process information by learning patterns from data. In computer-based data mining, they have found extensive use in many domains. The effectiveness of pattern analysis has been significantly improved through the application of ANNs in biological data mining. As an extended form of ANN, Deep Neural Networks (DNNs) allow for the exploration of more complex architectures and the capability to address intricate patterns and relationships within large datasets. There are two characteristics shared by the majority of neural network-based algorithms. As a first point, it uses a network of interconnected artificial neurons. Learned information from a dataset is subsequently represented by these model parameters, which are the links between the various components. Due to this characteristic, neural network models resemble the human brain. Secondly, when learning, a DNN model usually does not assume anything about the distribution of the data. DNNs are made significantly more applicable to a wide range of applications as a result of this [48].

### 3.6. Balancing Datasets

Balancing datasets is crucial in machine learning, particularly when dealing with imbalanced data where one class significantly outnumbers others [49]. Such imbalances can lead models to become biased toward the majority class, resulting in poor performance for the minority class, which is often of greater interest in applications like healthcare. We chose the Synthetic Minority Oversampling Technique (SMOTE) as our primary imbalance mitigation method. SMOTE was selected over alternative approaches such as random oversampling, undersampling, and class-weighting for several reasons: (i) Unlike random oversampling, SMOTE generates synthetic samples rather than duplicating existing ones, which helps reduce the risk of overfitting. (ii) In contrast to undersampling, SMOTE preserves the information contained in the majority class, which is crucial given the moderate dataset size. (iii) While class-weighting is supported by some algorithms, it is not universally applicable, and in preliminary experiments, it did not yield consistent performance improvements across models. Additionally, hybrid approaches combine both strategies for better results. Advanced machine learning models, such as ensemble methods like Random Forest and Gradient Boosting, can inherently handle class imbalances by assigning weights to classes. Cost-sensitive learning introduces penalties for misclassifying the minority class, encouraging models to focus on it. By using these strategies, practitioners can ensure more robust and fair model performance across all classes.

SMOTE is generally preferred for balancing datasets because it effectively addresses class imbalances without simply duplicating existing samples [50]. Unlike traditional oversampling, which duplicates minority class instances and risks overfitting, SMOTE generates synthetic samples by interpolating between existing minority class data points. In practical scenarios, datasets often exhibit significant class imbalances, where one class (typically the minority class) is underrepresented compared to others. Therefore, mastering techniques like SMOTE is critical for training robust models capable of handling real-world datasets.

**Figure 8:**
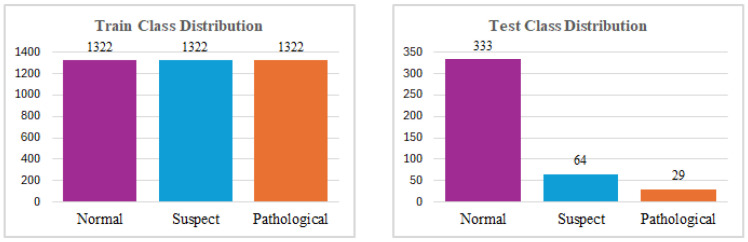
Training and Testing Dataset Distributions After SMOTE.

SMOTE tackles the issue of class imbalance by generating synthetic samples for the minority class through a process known as oversampling. Unlike simple duplication of existing data points, SMOTE creates new synthetic samples by interpolating between a data point from the minority class and one of its nearest neighbors within the same class. This is achieved by considering a linear combination of the feature vectors of the selected data point and its neighbor, thus producing realistic and diverse synthetic samples [51,52]. Each minority data sample contributes to the generation of a comparable number of synthetic samples, ensuring a balanced representation of classes in the dataset. Using this approach, SMOTE improves the model’s ability to learn patterns from the minority class, leading to improved predictive performance and more equitable outcomes in ML tasks. Subsequently, synthetic data was produced using the SMOTE method on the train dataset due to class inequality, and the equalized distribution was shown in Figure 8. The data prepared for training was processed by each ML algorithm, and model metrics (accuracy, precision score, f1-score, and recall value) were calculated.

**Figure 9:**
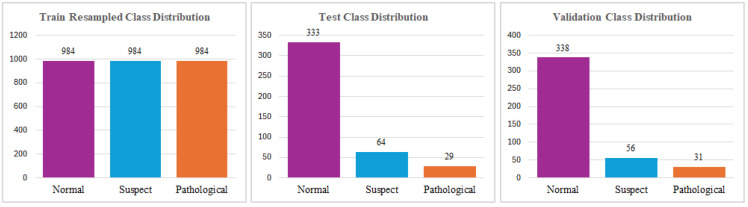
Training, Testing and Validation Dataset Distributions after SMOTE.

For the implementation of the ANN algorithm, the dataset was first divided into two subsets, with 80% allocated for training and 20% for testing. Subsequently, 25% of the training dataset was further separated to serve as a validation set, ensuring that the model could be evaluated on unseen data during the training process. To address the issue of class imbalance present in the dataset, the SMOTE was applied exclusively to the training set, as illustrated in Figure 9. By generating synthetic samples for the minority class, SMOTE enhanced the representation of the underrepresented class, thereby mitigating the bias that may arise due to class inequality. This pre-processed and balanced dataset was then used for training the ANN model, ensuring improved generalization and performance during the testing phase.

## 4. Experimental Results

In this section, the performance of the proposed fetal health classification system is evaluated using 1 Deep Learning algorithm as DNN and 8 ML algorithms such as DT, RF, KNN, Extra Trees, SVM, Gradient Boosting, LightGBM, and CatBoost. These algorithms were chosen to provide a comprehensive comparison of various classification techniques, ranging from traditional methods to advanced ensemble models, in terms of their ability to accurately classify fetal health status. Fetal health was predicted using the CTG dataset and evaluated the performance metrics of each. The experiments were conducted using the Python programming language and various ML libraries as depicted in Table 3.

Performance metrics for each algorithm were calculated following the training process on the balanced dataset created using SMOTE. The SMOTE process ensured that the class distribution within the dataset was balanced, allowing the algorithms to perform more effectively in classifying minority classes without bias.

To comprehensively evaluate the models, performance metrics such as accuracy, recall, and error rate were calculated individually for each class within the dataset. These class-specific metrics provide information on how well each algorithm performs in all categories, particularly in identifying instances of underrepresented classes. Subsequently, the individual class metrics were aggregated into overall scores using weighted averages, ensuring that the contribution of each class to the final metric is proportional to its size within the dataset. The formulas used for these calculations are detailed in Equations (1)–(4).

(1) $$Accuracy=\frac{Correct\;Predictions}{All\;Predictions}$$

(2) $$Recall_{i}=\frac{True\;Positive_{i}}{True\;Positive_{i}+False\;Negative_{i}}$$

(3) $$Balanced\;Accuracy=Recall_{Macro\;Avg}=\frac{Recall_{1}+Recall_{2}+Recall_{3}}{Total\;Number\;of\;Classes}$$

(4) Balanced Error Rate=1−Balanced Accuracy

Table 4 presents a comparative evaluation of several machine learning models based on their accuracy and balanced accuracy, assessed using both a split train-test 80–20% and a five-fold cross-validation because it plays a vital role in machine learning by offering a more reliable assessment of model performance than a single random train-test split. The analysis emphasizes the stability of standard accuracy and the notable improvements in balanced accuracy achieved through cross-validation. Across all models, standard accuracy exhibited minimal variation between the train-test split and cross-validation evaluations. LightGBM consistently achieved the highest accuracy (95.67% for the train test and 95.16% for cross-validation), followed by CatBoost (95.25% and 94.97%, respectively) and Gradient Boosting (94.64% and 93.84%, respectively). These results suggest that standard accuracy remains relatively stable regardless of the evaluation strategy, reflecting similar performance in correctly classifying the majority of instances.

In contrast, balanced accuracy showed significant improvement with cross-validation. This improvement is particularly critical for imbalanced datasets, as balanced accuracy evaluates performance across all classes rather than being biased toward the majority class. Among the models, SVM demonstrated the most notable improvement in balanced accuracy, increasing from 74.48% (train-test) to 82.01% (cross-validation), representing a 10.11% improvement. Similarly, KNN exhibited a substantial gain, with its balanced accuracy rising from 76.76% to 83.32%, an increase of 8.55%. These results highlight the sensitivity of these models to dataset partitioning and their improved generalization capability with cross-validation.

The findings suggest that standard accuracy alone may not provide a comprehensive view of model performance, especially for imbalanced datasets. Although the standard accuracy remained stable, the considerable improvements in balanced accuracy underscore the importance of cross-validation as a robust evaluation strategy. Models like SVM and KNN, which initially underperformed, showed remarkable enhancement, demonstrating the effectiveness of cross-validation in mitigating the challenges of class imbalance.

In general, the results highlight the need to prioritize balanced accuracy over standard accuracy for a more nuanced and reliable assessment of model performance in imbalanced scenarios. Therefore, balanced accuracy is adopted as the primary metric for ongoing performance evaluations.

Table 5 presents a comparative analysis of various machine learning models based on their balanced accuracy, error rates, and the improvements observed when SMOTE was applied. These findings provide valuable information on the performance of the models and highlight the significant impact of SMOTE on the handling of unbalanced datasets. In the baseline evaluation (without SMOTE), LightGBM emerged as the most effective model, achieving the highest balanced accuracy (90.93%) and the lowest error rate (9.07%). Other models, such as CatBoost and Gradient Boosting, also demonstrated strong performances, with balanced accuracies of 89.67% and 89.42%, respectively, accompanied by relatively low error rates. To address the effects of dataset imbalance, SMOTE was applied, and as shown in the table, it had a considerable positive impact across all models by improving the balanced accuracy and reducing the error rates. The most significant reduction in error rate was observed for SVM, which decreased from 25.52% to 17.99%, reflecting an improvement of 29.50%. Similarly, KNN showed a substantial improvement, with its error rate reduced by 28.23%. These enhancements underscore the effectiveness of SMOTE in mitigating class imbalance, especially for models that initially struggled in the baseline evaluation.

Post-SMOTE, LightGBM maintained its position as the leading model in balanced accuracy, with Gradient Boosting following closely behind. Although SVM showed notable improvement, it remained one of the weaker performers in absolute terms. CatBoost and Random Forest continued to perform consistently, while KNN and Decision Tree showed significant improvement, but still lagged behind the top-performing models. To better understand the impact of SMOTE, it is helpful to examine the average improvement across performance metrics. In this analysis, the average error rate reduction was measured at 19.13%, highlighting the utility of SMOTE in improving the robustness of the model when dealing with imbalanced datasets. This average gain demonstrates the clear advantages of incorporating SMOTE into the preprocessing pipeline for classification tasks where class imbalance poses a significant challenge.

Training a Deep Neural Network (DNN)-based model (as an example of Deep Learning Model) for classification is essential when traditional models struggle to capture complex, non-linear patterns in the data. DNNs excel in automatically learning features and hierarchies from raw data, making them suitable for high-dimensional datasets such as images, text, or genomic data. Unlike traditional models, which often require extensive feature engineering, DNNs adapt autonomously to complex and diverse data types, including images and sequences, without manual intervention. They are also better equipped to handle large datasets, where they continue to improve in performance, and imbalanced datasets, by leveraging advanced techniques like class weighting or custom loss functions. Additionally, DNNs provide flexibility in architecture and scalability, with specialized variants such as CNNs and RNNs tailored for specific tasks. They include advanced regularization methods, such as dropout and batch normalization, to prevent overfitting and ensure robust generalization. By offering superior performance on complex datasets and emerging tasks in fields like healthcare and autonomous systems, DNNs often outperform traditional models. Comparison of DNNs with traditional approaches also enables benchmarking, providing valuable insights into the best solution for the problem at hand. As we mentioned previously, we divided the dataset applied to SMOTE into three: test, train, and validation.

Therefore, in this paper, we also aimed to construct a neural network structure to evaluate the performance of the system using a deep learning approach, focusing on designing a robust architecture that could effectively handle complex data patterns. This involved selecting appropriate hyperparameters, such as the number of layers, activation functions, and learning rates (as depicted in Table 6), to optimize the accuracy and reliability of the model. Additionally, the model was intended to be trained and tested on relevant datasets to assess its capability for real-world applications and its potential for scalability in similar tasks.

As shown in Table 7, the performance of the models in the training, validation, and test sets were compared using standard accuracy and balanced accuracy, evaluated with a split of the train-test 80–20% and 5-fold cross-validation. The results reveal that while standard accuracy remains largely unchanged, balanced accuracy shows substantial improvements, particularly for the validation and test sets when cross-validation is employed with traditional machine learning models.

The standard accuracy remained consistent between the train-test split and the cross-validation methods. For the training set, there was a slight decrease in accuracy, from 96.62% to 95.84%, while the test set maintained the identical accuracy of 91.08% across both evaluation strategies. In contrast, balanced accuracy demonstrated significant gains. For the validation set, balanced accuracy increased notably from 76.06% (train-test) to 86.24% (cross-validation), reflecting a substantial enhancement of 13.38%. Similarly, the test set’s balanced accuracy improved from 81.07% to 85.06%, representing a 4.92% increase. These findings highlight the effectiveness of cross-validation in improving model evaluation in imbalanced datasets by providing a more generalized performance assessment.

The analysis underscores that, while the standard accuracy remains stable across evaluation methods, balanced accuracy benefits significantly from cross-validation, especially for validation and test sets. These improvements emphasize the importance of cross-validation as a robust evaluation approach for imbalanced datasets, enabling better generalization and more equitable performance across all classes. Consequently, balanced accuracy should be prioritized as a critical metric for assessing model performance in such scenarios. Furthermore, the results demonstrate the impact of the SMOTE method when applied in a deep learning framework, such as DNN.

Deep learning approaches are anticipated to outperform traditional machine learning models in future research, particularly when applied to larger and more diverse datasets. While conventional models tend to perform well on smaller datasets due to their lower complexity and reduced risk of overfitting, deep learning models have the potential to uncover more complex patterns and subtle relationships within the data—capabilities that become increasingly advantageous as dataset size grows.

**Figure 10:**
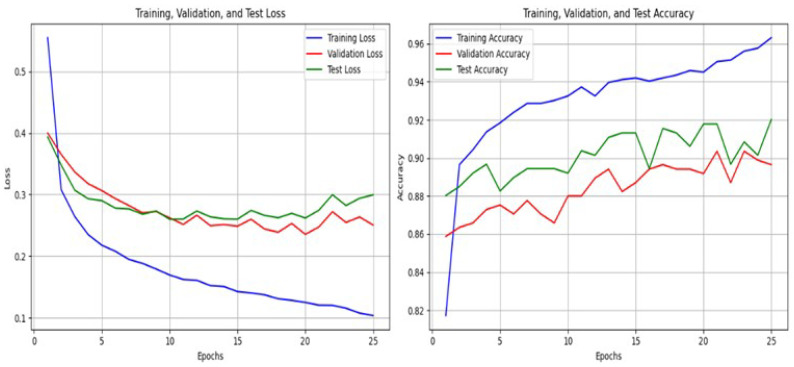
Accuracy and Loss Graphs for DNN.

Figure 10 illustrates the progression of training, validation, and test accuracy over 25 epochs during the training process of a machine learning model. By analyzing the trends and relationships between these metrics, several key insights can be derived. The training accuracy, depicted by the blue curve, shows a steady increase as the epochs progress. This upward trend indicates that the model is effectively learning from the training data, reaching nearly 96% accuracy by the final epoch. The sharp increase in accuracy during the initial epochs, followed by a more gradual improvement, suggests that the model quickly identifies basic patterns in the data before fine-tuning its performance in subsequent epochs. This steady improvement implies that the model is successfully fitting the training data. In contrast, the validation accuracy, represented by the red curve, initially increases but exhibits fluctuations throughout the training process. These fluctuations indicate that, while the model improves its performance on unseen data, it also encounters variability, likely due to overfitting or the complexity of the data. A noticeable gap between training and validation accuracy emerges after the 10th epoch, suggesting that the model begins to overfit the training data as it continues to optimize.

The test accuracy, shown by the green curve, closely mirrors the trend of the validation accuracy. After the initial epochs, it stabilizes around 91% with minor fluctuations. The similarity between the test and validation accuracy curves indicates that the model generalizes well to unseen data, and the validation set effectively represents the test set. This consistency reinforces the reliability of the model performance evaluation. Overall, the figure demonstrates that the model achieves strong performance, with high accuracy across all metrics. The steady improvement in training accuracy, combined with the stable trends in validation and test accuracy, highlights the model’s ability to learn from the data while maintaining good generalization to unseen samples.

Studies focusing on fetal and maternal health are of paramount importance in the ongoing effort to reduce fetal and maternal mortality rates, which remain critical indicators of healthcare quality worldwide. Advances in AI have introduced transformative possibilities in this domain, as evidenced by numerous studies that demonstrate AI’s ability to expedite the evaluation of fetal health and enhance decision-making processes for clinicians. These systems provide not only a rapid assessment of critical parameters, but also a consistent and objective approach that complements the expertise of healthcare professionals.

In our study, we used CTG data, a standard diagnostic tool in obstetrics, to train and evaluate 8 ML algorithms and a DNN algorithm (as a Deep Learning model), each offering unique strengths and limitations. Among these, the LightGBM algorithm emerged as the most effective, achieving superior accuracy and reliability in classifying the fetal health status. Based on these findings, a user-friendly interface was developed that incorporates the LightGBM model, enabling healthcare providers to seamlessly input CTG data and receive rapid diagnostic feedback. This interface bridges the gap between advanced AI technologies and practical clinical application, making the tool accessible and intuitive for medical practitioners.

Traditional monitoring methods often rely on the manual interpretation of cardiotocography (CTG) by clinicians, a process that can be subjective, time-consuming, and susceptible to inter-observer variability. In contrast, our proposed machine learning-based approach offers several significant advantages. First, it provides objective decision support by delivering automated and consistent evaluations of fetal health, thereby reducing dependence on subjective clinical judgment and enhancing diagnostic reliability. Second, the system is capable of early risk detection by identifying subtle patterns and early indicators of fetal distress through advanced algorithms trained on historical CTG data—patterns that may be overlooked during manual assessments. Additionally, the model’s automated nature allows for real-time analysis and scalability across diverse healthcare settings, which is particularly beneficial in resource-limited environments where access to expert obstetricians may be constrained. Finally, integrating this system into clinical workflows can support healthcare professionals in prioritizing high-risk cases and optimizing intervention strategies, ultimately contributing to improved maternal and neonatal outcomes.

The proposed model can be integrated into the clinical workflow as a decision support system within existing fetal monitoring infrastructure. Specifically, it could be embedded into electronic fetal monitoring (EFM) systems or hospital information systems (HIS), where it would automatically process cardiotocography (CTG) data in real time and provide clinicians with risk-level assessments (e.g., normal, suspicious, or pathological). These outputs can be presented through a simple graphical interface, enabling quick and intuitive interpretation by healthcare professionals.

## 5. Conclusions

The study underscores the transformative potential of Machine Learning within the field of obstetrics, specifically for the classification of fetal health. By evaluating various Machine Learning models on a dataset derived from Cardiotocography data, the research highlights the LightGBM model as the most effective oane and achieving an impressive balanced accuracy rate of 91.34%. This finding not only showcases the superiority of the LightGBM model over other models such as the Random Forest, but also demonstrates the significant advances ML can bring to medical diagnostics. The integration of these models, particularly LightGBM, into clinical practice can streamline the analysis of fetal health data, providing rapid and reliable support to healthcare professionals. The development of a user interface based on the LightGBM model in this study represents a crucial step toward real-time application, suggesting that future implementations could see these systems directly linked to CTG devices. Such an integration would enable instantaneous results, thus enhancing the decision-making process for doctors and potentially improving maternal and fetal health outcomes.

In this study, the positive impact of SMOTE on the development of classification systems to address imbalanced datasets, specifically within the context of fetal health conditions, is thoroughly analyzed. The application of SMOTE significantly enhances the performance of machine learning models by incrementally improving balanced accuracy, a critical metric that emphasizes the accurate detection of minority classes associated with severe fetal health risks. This improvement is particularly vital in fetal diagnosis, where accurately identifying minority conditions is crucial for effective intervention and treatment, thus ensuring equitable diagnostic performance in all fetal health categories.

Furthermore, the study examines the impact of a Deep Learning model, structured as a two-layer Deep Neural Network, specifically on classifying fetal health conditions. The experimental findings highlight that, given the constraints of limited medical datasets, the proposed Deep Learning models do not surpass traditional machine learning methods in accurately identifying fetal health problems. This emphasizes significant limitations in the use of deep learning approaches within small-scale medical datasets, where inadequate data impede the model’s ability to effectively generalize and detect intricate patterns crucial for the diagnosis of fetal health.

Future studies could focus on exploring this potential by utilizing larger datasets and further optimizing the architecture of the Deep Learning models to fully exploit their advantages in handling complex and high-dimensional data. Additionally, extending the developed user interface into a fully functional, end-to-end platform, complete with cloud-based capabilities for data storage and analysis, could facilitate broader adoption. The platform could be designed to incorporate continuous learning, allowing it to adapt and improve as more data become available.

## Figures and Tables

**Table 1:** Comparison of Related Studies.

Authors	Year	Dataset	Methods Used	Classification	Best Model and Outcome	Solving Imbalanced Dataset—Method
Rahmayanti et al. [15]	2022	UCI	ANN, LSTM, XGB, SVM, KNN, LGBM, RF	Multiclass	LGBM, AUC = 99.00%, F1-score = 98.00%	Yes—Sampling
Li et al. [16]	2021	UCI	Gradient Boosting, CatBoost, Light Gradient Boosting, Cascade Forest, AdaBoost	Multiclass	Blender Model, AUC = 98.8%, F1-score = 95.80%, Acc. = 95.90%, Rec. = 91.60%, Pre. = 95.90%, MCC = 88.60%	No
Miao et al. [17]	2018	UCI	Deep Neural Network	Multiclass	DNN, Acc. = 88.02%, Rec. = 84.30%, Pre. = 85.01%, F1-score = 85.08%	No
Kuzu et al. [18]	2023	UCI	GB, LR, XGBoost, RF	Multiclass	XGBoost, 99.50%	No
Regmi et al. [19]	2023	UCI	Deep Learning, TabNet, SVM, RF	Multiclass	TabNet, 94.36%	No
Kapila et al. [20]	2023	UCI	LR, DT, RF, KNN, NB, SVM, MLP	Multiclass	SMOTE with Chi2-PCA using SC with LG metaclassifier, 98.79%	Yes—SMOTE
Mandala [21]	2024	UCI	LGBM, CatBoost, ET, XGB, RF, GBC, DT, KNN, ADA, LR, RIDGE, SVM, LDA, QDA, NB	Multiclass	LightGBM, Acc. = 97.91%	Yes—SMOTE
Brzoza et al. [22]	2021	UCI	KNN, NB, Softsets	Multiclass	KNN for k = 1, Acc. = 90.61%	No
Signorini et al. [23]	2019	UCI	RF, SVM, CT, NB, LR	Multiclass	RF, Acc. = 91.10%	No
Comert et al. [24]	2016	UCI	ANN, SVM, ELM, RBFN, RF	Multiclass	ANN, Sensitivity = 99.73%, Specificty = 97.94%	No
Dadario et al. [25]	2021	UCI	RF, LGBM, CVE LGBM	Multiclass	CVE LGBM, Acc. = 95.14%, F1-score = 91.28%, Rec. = 89.86%, Pre. = 92.86%	No
Alam et al. [26]	2022	UCI	RF, DT, SVM, KNN, LR, VC	Multiclass	Random Forest, Acc. = 97.51%	Yes—SMOTE
Rayhan et al. [27]	2021	UCI	RF, LR, SVM, KNN, XGBoost	Multiclass	XGBoost, Acc. = 96.70%, F1-score = 96.30%	No
Talukder et al. [28]	2023	UCI	SVM, XGB, LGBM, DT, RF, ET, KNN, Ensemble Learning	Multiclass	ETSE (ET + SVM), 99.66%	No
Das et al. [29]	2023	CTU-UHB	RF, MLP, SVM, Bagging	Multiclass	RF, Sensitivity = 96.40%, Specificity = 98.40%	Yes—SMOTE
Pradhan et al. [30]	2021	UCI	LR, KNN, RF, GBM	Multiclass	RF, Acc. = 99.00%	No
Islam et al. [31]	2023	UCI	AdaBoost, RF, KNN, SVM, GBC, DT, LR	Multiclass	GBC, Acc. = 95.00%, Pre. = 100%	No
Yin et al. [32]	2023	UCI	LightGBM, XGBClassifier, SVM with FAB	Multiclass	SVM, Acc. = 99.59%	Yes—Oversampling
Dwivedi et al. [33]	2021	UCI	LightGBM, GBM, RF, ETC	Multiclass	LGBM, Acc. = 95.61%, Rec. = 90.56%, AUC = 98.64%, F1-score = 95.64%	Yes—SMOTE

**Table 2:** Description of CTG Dataset.

Attribute	Description and Units	Mean	Std	Min	Max
LB	Fetal Heart Beats per minute	133.30	9.84	106	160
AC	Accelerations per second	$3.1\times 10^{-3}$	$3.8\times 10^{-3}$	0.0	0.019
FM	Fetal movements per second	$9.4\times 10^{-3}$	$4.6\times 10^{-2}$	0.0	0.481
UC	Number of uterine contractions per second	$4.3\times 10^{-3}$	$2.94\times 10^{-3}$	0.0	0.015
LD	Number of light decelerations per second	$1.8\times 10^{-3}$	$2.96\times 10^{-3}$	0.0	0.015
SD	Number of severe decelerations per second	$3\times 10^{-6}$	$5.7\times 10^{-5}$	0.0	0.001
PD	Number of prolonged decelerations per second	$1.59\times 10^{-4}$	$5.9\times 10^{-5}$	0.0	0.005
ASTV	Percentage of time with abnormal short-term variability	46.9	17.19	12.0	87.0
MSTV	Mean Value of Short Term Variability	1.33	0.88	0.2	7.0
ALTV	Percentage of Time with Abnormal Long-Term Variability	9.84	18.39	0.0	91.0
MLTV	Mean Value of Long-Term Variability	8.18	5.62	0.0	50.7
HW	Width of FHR histogram	70.44	38.95	3.0	180.0
HM	Maximum of FHR histogram	164.02	17.94	122.0	238.0
HMin	Minimum of FHR histogram	93.57	29.56	50.0	159.0
NP	Histogram Peaks	4.06	2.94	0.0	18.0
NZ	Histogram Zeroes	0.32	0.70	0.0	10.0
HMo	Histogram Mode	137.45	16.38	60.0	187.0
HMe	Histogram Mean	134.61	15.59	73.0	182.0
HMed	Histogram Median	138.09	14.46	77.0	186.0
HV	Histogram Variance	18.80	28.97	0.0	269.0
HT	Histogram Tendency	0.32	0.61	−1.0	1.0
NSP	Fetal state class code, (0: normal (N); 1: Suspect (S); 2: Pathological (P))	0.30	0.61	0.0	2.0

**Table 3:** Libraries and tools used in the analysis.

Library/Tool	Purpose
Pandas	Used for data loading and data frame manipulation.
NumPy	Used for numerical calculations and data processing.
Scikit-learn	Used for the creation, training, and evaluation of ML models. Performance metrics are also calculated with this library.
Imbalanced learning	The SMOTE algorithm was used to correct class imbalances in the dataset.

**Table 4:** The performance metrics with traditional data division and 5-fold cross validation.

	80% Train, 20% Test		Cross Validation (5 Fold)		
**Model**	**Acc. (%)**	**Bal. Acc. (%)**	**Acc. (%)**	**Bal. Acc. (%)**	**Bal. Acc. Inc. (%)**
CatBoost	95.25	89.67	94.97	91.00	1.49
Decision Tree	90.55	79.68	88.33	84.13	5.59
ExtraTrees	93.04	83.91	93.74	87.88	4.74
Gradient Boosting	94.64	89.42	93.84	91.04	1.81
KNN	89.46	76.76	86.36	83.32	8.55
LightGBM	95.67	90.93	95.16	91.34	0.45
Random Forest	94.31	87.14	93.70	89.19	2.36
SVM	87.86	74.48	83.40	82.01	10.11
Average					4.16

**Table 5:** Performance Improvements of the model after using SMOTE.

Model	Bal. Acc. (%)	Bal. Err. Rate (%)	Bal. Acc. (SMOTE) (%)	Bal. Err. Rate (SMOTE) (%)	Bal. Err. Rate Dec. (%)
CatBoost	89.67	10.33	91.00	9.00	12.89
Decision Tree	79.68	20.32	84.13	15.87	21.92
ExtraTrees	83.91	16.09	87.88	12.12	24.72
Gradient Boosting	89.42	10.58	91.04	8.96	15.30
KNN	76.76	23.24	83.32	16.68	28.23
LightGBM	90.93	9.07	91.34	8.66	4.54
Random Forest	87.14	12.86	89.19	10.81	15.97
SVM	74.48	25.52	82.01	17.99	29.50
Average					19.13

**Table 6:** Experimental Parameters for DNN.

Parameter	Value
1st Layer Neurons	128
2nd Layer Neurons	64
Epochs	50
Batch Size	32
Learning Rate	0.0005
Activation Function (1st Layer)	Tanh
Activation Function (2nd Layer)	Sigmoid
Activation Function (Output Layer)	Softmax

**Table 7:** The performance metrics before and after SMOTE for DNN model.

	80% Train, 20% Test		Cross Validation (5 Fold)		
**Dataset**	**Acc. (%)**	**Bal. Acc. (%)**	**Acc. (%)**	**Bal. Acc. (%)**	**Bal. Acc. Inc. (%)**
Training	96.62	94.06	95.84	92.19	−1.99
Validation	89.41	76.06	91.76	86.24	13.38
Test	91.08	81.07	91.08	85.06	4.92

## Data Availability

The original data presented in the study are openly available in Kaggle at https://www.kaggle.com/datasets/andrewmvd/fetal-health-classification, accessed on 14 January 2025.
